# The Temperature-Sensitive Anisotropic Negative Poisson’s Ratio of Carbon Honeycomb

**DOI:** 10.3390/nano9040487

**Published:** 2019-03-28

**Authors:** Wenrui Wang, Chenwei He, Lu Xie, Qing Peng

**Affiliations:** 1 School of Mechanical Engineering, University of Science and Technology Beijing, Beijing 100083, China; gmbitwrw@ustb.edu.cn; 2 China Nuclear Power Technology Research Institute Co., Ltd., Reactor Engineering and Safety Research Center, Shenzhen 518031, China; hechenwei@cgnpc.com.cn; 3 Nuclear Engineering and Radiological Sciences, University of Michigan, Ann Arbor, MI 48108, USA

**Keywords:** carbon honeycomb, molecular dynamics simulations, negative Poisson’s ratio, auxiticity

## Abstract

We report that carbon honeycomb, a new three-dimension carbon allotrope, exhibits large negative Poisson’s ratio, as large as −0.32, in tensile revealed via molecular dynamics simulations. The Poisson’s ratio of carbon honeycomb is anisotropic, and sensitive to temperature. The carbon honeycomb has phase transformation from normal to auxetic by tensile, along both zigzag and armchair directions. The critical strain for the normal-auxetic transition along the cell-axis direction reduces with respect to an increase in temperature. Combined with high strength of 50 GPa, such a unique and adjustable negative Poisson ratio suggests broad engineering applications of carbon honeycomb.

## 1. Introduction

When a solid is stretched in one direction, it usually shrinks in the other two directions orthogonal to the applied loading. Such a change in shape is quantified by the parameter, Poisson’s ratio, which is defined as the ratio of transverse contraction to the longitudinal extension in the stretching direction. Most solids exhibit positive Poisson’s ratio (PPR), known as normal materials. Some solids possess negative Poisson’s ratio (NPR), known as auxetic materials [1]. Auxetic materials could be two-dimensional (2D) or three-dimensional (3D). The 2D auxetic materials [2] include honeycombs, graphene, selected laminates, origami, and membranes. Some anisotropic single crystals and lattices are 3D auxetic materials [3,4]. With the unique feature of negative Poisson’s ratio, auxetic materials rise to be a new class of engineering materials, with applications in biomedical devices, aerospace engineering, and automotive engineering [5,6].

Extensive efforts have been devoted to discover NPR materials, from both fundamental understandings and engineering applications [7]. Wojciechowski proposed that 2D systems of both hard-cyclic hexamers [8] and hexagonal molecules on a triangular lattice [9] can display a negative Poisson’s ratio. Du et al. [10] found that there exists a NPR phenomenon in the low-dimensional natural material, black phosphorus, via both experimental studies and numerical simulations. With the same puckered atomic configuration as black phosphorus, orthorhombic arsenic exhibits NPR [11]. Zachary et al. [12] found that self-avoiding 2D membranes exhibit desirable auxetic properties over a range of mechanical strain. Similarly, graphene [13] has been reported to exhibit NPR at strain larger than 0.06 [14,15]. For carbon nanotube, the Poisson’s ratio is positive. However, it is reported that carbon nanotube sheets or films have auxetic properties [16,17], and Hall et al. [18] gained carbon nanotube sheets with NPR by mixing single-walled and multi-walled nanotubes.

Low-dimensional carbon allotropes have advanced properties due to the quantum confinement, including ultra-high strength, such as 1D carbon nanotubes [19,20] and 2D graphene [21,22]. It is a grand challenge to design 3D carbon materials, without much loss of the advanced properties as low-dimensions, as such properties are significantly lower than those of single graphene or CNTs when scaling up to a 3D structure [23,24]. The carbon honeycomb (CHC), with high stability and mechanical properties [25,26,27], could be a game-change player. CHC was firstly proposed by first-principles calculations in 2000 [28], followed by successful synthesis in 2014 [29]. With the existence of auxiticity in low-dimensions as a graphene and carbon nanotube sheet, we hypothesize that the properties of auxiticity could be preserved in the 3D carbon honeycomb. In this work, we validate this hypothesis via classic molecular dynamics simulations. We found that CHC has mechanical phase transitions from normal to auxetic (NA transition) at large strains. Such auxeticity is anisotropic and temperature dependent.

## 2. Computational Details

**Figure 1:**
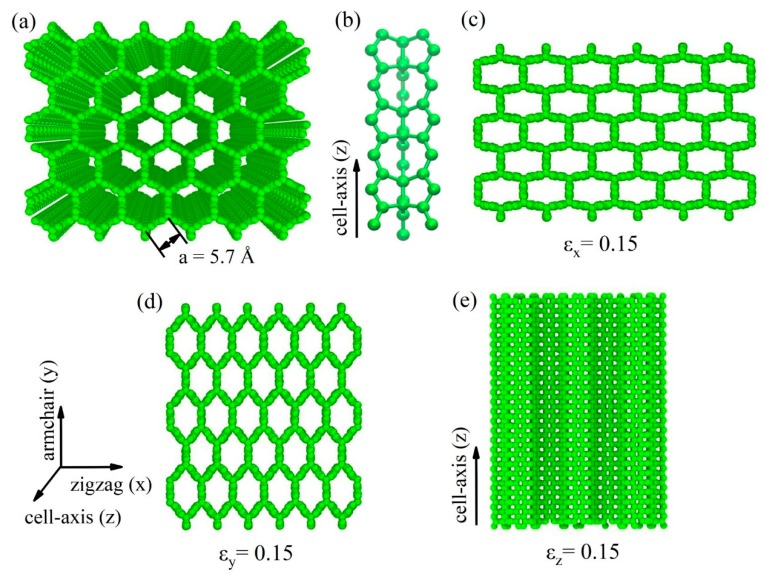
Stable carbon honeycomb structure. (**a**) Atomistic structure of carbon honeycomb. (**b**) Local atomic structure at the 5-5-8 junction of carbon honeycomb. (**c**–**e**) Snapshots of carbon honeycomb at 0.15 tensile strain along x, y, and z directions, respectively.

A stable 3D carbon honeycomb with sp^2^ bonding in the wall and sp^3^ bonding in the junction was constructed, as shown in Figure 1a. It consisted of zigzag-edged graphene nanoribbons. The cross-section perpendicular to the cell axis was a honeycomb structure based on a regular hexagon with the size a = 5.7 Å. The junction (Figure 1b) was composed of an array of cell units containing two 5-rings and one 8-ring, so it could be seen as a 5-5-8 junction [24]. The dimensions of the CHC structure were 59.43 Å × 51.51 Å × 57.87 Å.

Molecular dynamics (MD) simulations were employed to reveal the NA transition by the Large-scale Atomic/Molecular Massively Parallel Simulator (LAMMPS) package [30]. The atomic interactions for C–C were described by the adaptive intermolecular reactive empirical bond order (AIREBO) potential [31]. All MD simulations were simulated at the isothermal-isobaric ensemble (NPT), with periodic boundary conditions in all the directions. The Velocity-Verlet algorithm was employed to integrate Newton’s equations of motion with a time step of 0.5 fs. The temperature and pressure were controlled using a Nose Hoover thermostat and barostat, with coupling times set at 0.025 fs and 0.25 fs, respectively.

The geometry optimization of the atomic structure of CHC was performed using a conjugate gradient algorithm for a minimum potential energy first. Then a stable CHC structure with 12,906 atoms was obtained by relaxing at room temperature (300 K) and zero external pressure for 25 ps. The tensile tests with the CHC structure at 300 K were performed by expanding the box size along three directions (zigzag, armchair, and cell-axis directions, respectively) at engineering strain rate of 10^9^ s^−1^. Similarly, the temperature effect was also investigated for the temperature range of 200 K to 500 K.

## 3. Results and Discussion

### 3.1. Tensile Tests

The CHC showed higher tensile strength and bigger ultimate tensile strain along the cell-axis direction as the temperature decreased. As our previous work illustrated [32], ultimate tensile strength of CHC was about 23 GPa in the zigzag direction and 22 GPa in the armchair direction at 300 K. The comparison of the stress–strain curves of CHC from this work and DFT calculations [24] is plotted in Supplementary Figure S1 with the same scale of axis. Results obtained from MD simulations were slightly smaller than that obtained from DFT calculations. This is because MD simulations were carried out at the temperature of 300 K. The tensile strength along the cell-axis direction exceeded 50 GPa, exhibiting perfect mechanical property. Different deformation modes (as shown in Figure 1c–e) of CHC in three directions explained the anisotropic mechanical property. Engineering stress–strain curves of CHC at different temperatures are illustrated in Supplementary Figure S2 for different directions.

**Figure 2:**
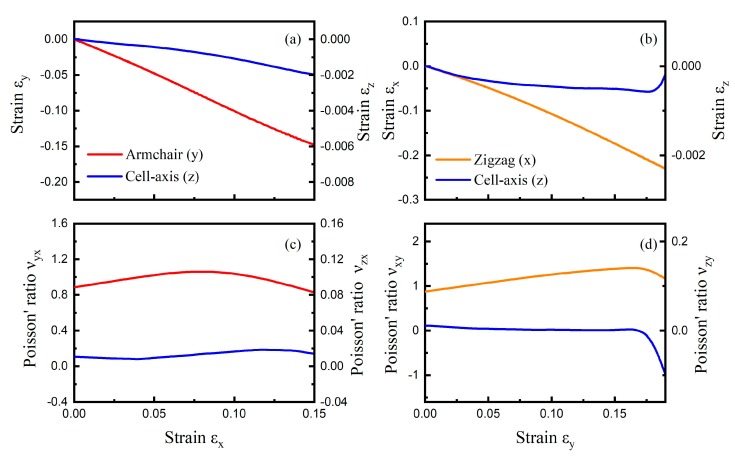
Normal to auxetic (NA transition) in carbon honeycomb (CHC). Engineering strain $\varepsilon_{y}$ and $\varepsilon_{z}$ versus $\varepsilon_{x}$ curves for CHC subjected to uniaxial tensile tests in the zigzag direction (**a**), and in the armchair direction (**b**). The accompanying ν for tensile tests in the zigzag direction (**c**), and armchair direction (**d**). The negative Poisson’s ratio is observed at about 0.168 strain when tensile strain was applied along armchair direction.

Considering the comparison stress–strain curves of CHC from MD (300 K) and DFT calculations in Supplementary Figure S1, we only cautiously report the results at small strain range (0.15 in x direction and 0.19 at y direction). The collateral strain and corresponding Poisson’s ratio as a function of tensile strain are shown in Figure 2 for tensile loading, along both zigzag and armchair directions, at the temperature of 300 K. The engineering strain $\varepsilon_{x}$ in the x direction is calculated by $\varepsilon_{x}=\left(L_{x}-L_{x0}\right)/L_{x0}$, with $L_{x0}$ and $L_{x}$ as the initial and deformed sample lengths in the x direction, respectively. The corresponding Poisson’s ratio is obtained by taking the first derivative of the transverse strain with respect to $\varepsilon_{x}$. Our results show that the NA transition occurred at about 0.168 strain along the cell-axis direction when the tensile strain was applied along the armchair direction (Figure 2b). This NA transition behavior was not observed in the zigzag direction (orange line in Figure 2b) or when tensile strain was applied along the zigzag direction (Figure 2a).

Tensile strain applied to the zigzag direction introduced nearly the same amount of compressive strain in the armchair direction (Figure 2a), which is in agreement with DFT calculations in literature [24]. $\nu_{\mathrm{yx}}$ was about 0.9 at zero strain. $\nu_{\mathrm{zx}}$ was around 0.013 at the tensile process, which is far less than $\nu_{\mathrm{yx}}$. The Poisson’s ratio of CHC varied with tensile strain, which is consistent to that of foam with NPR [33]. For tensile strain $\varepsilon_{y}$ up to 0.19 applied to the armchair direction of CHC, the strains $\varepsilon_{x}$ and $\varepsilon_{z}$ as a function of $\varepsilon_{y}$ are shown in Figure 2b, and the calculated Poisson’s ratios are shown in Figure 2d. It can be seen that $\varepsilon_{x}$ decreased from 0 to −0.22, and $\nu_{\mathrm{xy}}$ were around 1.0 when tensile strain $\varepsilon_{y}$ increased from 0 to 0.19. In general, the Poisson’s ratio along the armchair (or zigzag) direction was larger than that along the cell-axis direction when the tensile test was applied in the zigzag (or armchair) direction.

### 3.2. Temperature Effect for NA Transition

**Figure 3:**
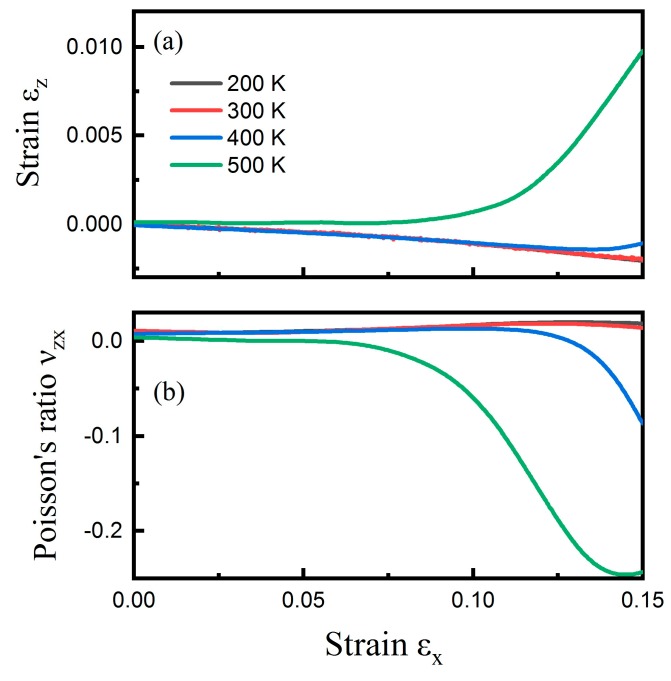
Temperature effect. The influence of temperature on the positive–negative Poisson’s ratio transition for CHC subjected to uniaxial tensile tests in the zigzag (x) direction. (**a**) Engineering strain $\varepsilon_{z}$ versus $\varepsilon_{x}$ at temperatures ranging from 200 to 500 K. (**b**) The corresponding $\nu_{\mathrm{zx}}$ for CHC at different temperatures.

The CHC structure exhibited negative Poisson’s ratio along the cell-axis direction. Furthermore, the temperature effect on the NA transition behavior was also investigated. In the temperature range of 200 to 500 K, the stress–strain curve in the x direction had little difference when the strain was less than 0.15, and the stress–strain curve in the y direction was almost the same when the strain was less than 0.19 (Supplementary Figure S2). Therefore, the temperature effect was studied in these strain ranges. Figure 3a shows the correlation between $\varepsilon_{z}$ and $\varepsilon_{x}$ up to 0.15 at the temperature range from 200 to 500 K when CHC was subjected to uniaxial tensile tests in the zigzag (x) direction. At 200 and 300 K, $\nu_{\mathrm{zx}}$ remained positive throughout the tensile test as $\varepsilon_{z}$ decreased with the increasing tensile strain. When the temperature increased, ranging from 400 to 500 K, the NA transition behavior was observed. There existed a critical temperature of 400 K, below which no NA transition occurred. Figure 3b shows $\nu_{\mathrm{zx}}$ as a function of tensile strain $\varepsilon_{x}$ at the four temperatures. The critical strain for NA transition reduced with respect to an increasing temperature. The critical strains were 0.128 (400 K) and 0.044 (500 K), respectively. Just like graphene sheets [34] and other 3D structures [35], the Poisson’s ratio of CHC showed to be highly strain dependent; it was slightly positive at a small tensile strain but became negative at a few percent strain. Particularly, the Poisson’s ratio as negative as −0.25 was achieved at 500 K in this case.

**Figure 4:**
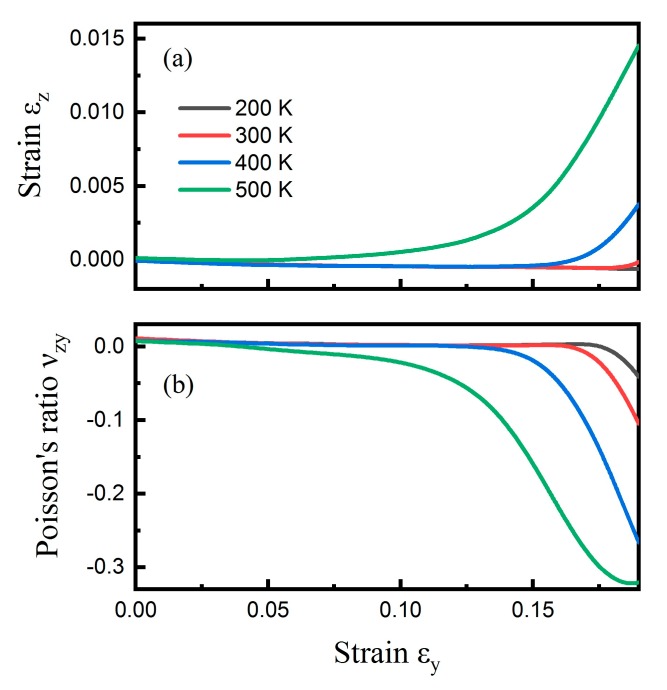
Temperature effect. The influence of temperature on the positive–negative Poisson’s ratio transition for CHC subjected to uniaxial tensile tests in the armchair (y) direction. (**a**) Engineering strain $\varepsilon_{z}$ versus $\varepsilon_{y}$ at temperatures ranging from 200 to 500 K. (**b**) The corresponding $\nu_{\mathrm{zy}}$ for CHC at different temperatures.

Regarding the armchair direction, Figure 4a shows the correlation between $\varepsilon_{z}$ and $\varepsilon_{y}$ up to 0.19, at the temperature range from 200 to 500 K, when CHC was subjected to uniaxial tensile deformation along the armchair (y) direction. It can be seen that higher temperatures induced a bigger transverse strain $\varepsilon_{z}$ at the same tensile strain $\varepsilon_{y}$. The $\varepsilon_{z}$ decreased slowly, and then increased with respect to an increasing tensile strain, indicating that there existed a NA transition. The corresponding Poisson’s ratios $\nu_{\mathrm{zy}}$ for CHC at different temperatures are shown in Figure 4b. At zero strain, $\nu_{\mathrm{zy}}$ were around 0.009, the same for all four temperatures. It decreased from positive to negative with the increase of tensile strain. The higher the temperature, the smaller the critical strain for the NA transition. It occurred at about 0.175, 0.168, 0.122, and 0.039 tensile strain for temperature of 200, 300, 400, and 500 K, respectively. A minimum Poisson’s ratio as small as approximately −0.32 was achieved at 500 K.

When tensile strain was applied along the cell-axis direction, the temperature effect on Poisson’s ratio was also investigated (as shown in Supplementary Figure S3). With the increasing temperature, the Poisson’s ratio $\nu_{\mathrm{xz}}$ at zero strain were around 0.14. At 300 K, $\nu_{\mathrm{xz}}$ decreased lineally, then turned to increase at strain 0.05. The maximum Poisson’s ratio was up to around 0.5. With increasing temperature, the maximum Poisson’s ratio occurred at a smaller tensile strain. When tensile strain was applied along the cell-axis direction in the y,z-plane, the same temperature effect was observed as in the x,z-plane. In addition, no NA transition phenomena was observed in the range of tensile strain $\varepsilon_{z}$ < 0.15.

It is worth noting that foams can be isotropic with Poisson’s ratio as small as −0.8, as reported in [2]. In this work, we report that CHC are anisotropic. The Poisson’s ratio can be tuned to be as small as −0.32 by tensile strains. It was reported that the NA transition for monolayer graphene has a much larger temperature range, from 50 to 2400 K [15]. While the NA transition for CHC is sensitive to temperature. Increasing temperature can reduce the critical strain where the Poisson’s ratio turns to negative. Therefore, the Poisson ratio of CHC are anisotropic and temperature dependent. Such special properties provide broad promising applications.

The auxeticity is highly desirable in some applications including impact mitigation, sealants, and water desalination. Therefore, auxetic materials have various applications in stents, skin grafts, smart bandage, artificial blood vessels, batting pads, smart sensors, and aero engine fan blades. Combined with high strength and stability, the discovery of NA transition for CHC could extend the applications of 3D carbon allotropes to new horizons.

## 4. Conclusions

In conclusion, we have investigated the normal-auxetic mechanical phase transition and tensile strength of CHC through classical molecular dynamics simulations. Our results show that the NA transition for CHC is anisotropic and temperature dependent. The Poisson’s ratio can be tuned to be as small as −0.32 by tensile strains. The critical strain (if applicable) for NA transition decreases with an increase in temperature. It is as small as 0.044 (tensile along zigzag direction) and 0.039 (armchair) at 500 K. The tensile strength of CHC along the cell-axis direction exceeds 50 GPa, exhibiting perfect mechanical property. The unique NA transition for CHC suggest the promising applications of 3D carbon allotropes in seawater desalination, impact absorbers, skin grafts, artificial blood vessels, smart sensors, aero engine fan blades, and other various engineering applications.

## Data Availability

The datasets generated and/or analyzed during the current study are available from the corresponding author on reasonable request.

## Supplementary Materials

The following are available online at https://www.mdpi.com/2079-4991/9/4/487/s1. The following files are available free of charge. Engineering stress–strain curves of CHC, and the influence of temperature on the Poisson’s ratio when tensile tests are applied along the cell-axis direction.
