# Supplementary material for: The Temperature-Sensitive Anisotropic Negative Poisson’s Ratio of Carbon Honeycomb
Source: Nanomaterials (Basel). 2019 Mar 28;9(4):487. doi: 10.3390/nano9040487 (PMC6523722; doi:10.3390/nano9040487)
Supplement: Supplementary file 1 [file nanomaterials-09-00487-s001.pdf]

Supplementary Information

The Temperature-Sensitive Anisotropic Negative Poisson Ratio of Carbon Honeycomb

Wenrui Wang[1], Chenwei He [2], Lu Xie [1,*] and Qing Peng [3,*]

[1] University of Science and Technology Beijing, School of Mechanical Engineering, Beijing 100083, China; gmbitwrw@ustb.edu.cn
[2] China Nuclear Power Technology Research Institute Co., Ltd., Reactor Engineering and Safety Research Center, Shenzhen 518031, China; hechenwei@cgnpc.com.cn
[3] University of Michigan, Nuclear Engineering and Radiological Sciences, Ann Arbor 48108, MI, USA

* Correspondence: xielu@ustb.edu.cn (L.X.), q@qpeng.org (Q.P.)

Supplementary Figures

The comparison of the stress-strain curves of CHC from this work and DFT calculations [1] is plotted in Supplementary Figure S1 with the same scale of axis. Results obtained from MD simulations are slightly smaller than that obtained from DFT calculations. In armchair and cell axis directions, stress-strain curves obtained from MD method show yield stage, which is different from DFT calculations. When tensile along cell axis direction, the yield stage corresponds to the break of C-C bonds on the junction of CHC. It is the fracture of C–C bonds on the junction that leads to the yield behavior in cell axis direction.

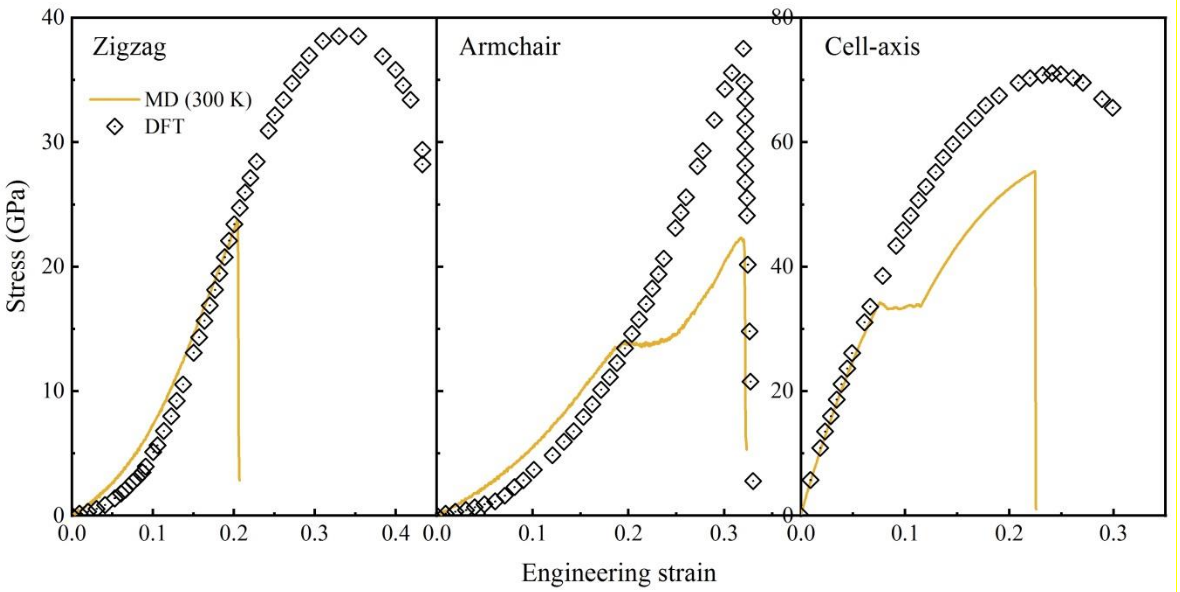

Figure S1. A comparison of engineering strain-strain curves of CHC from MD (300 K) and DFT calculations.

Supplementary Figure S2 shows the engineering stress-strain curves of CHC at different temperatures. The ultimate tensile strength of CHC is about 23 GPa in the zigzag direction and 22 GPa in the armchair direction. While the strength along cell-axis direction exceeds 50 GPa, which is two times larger than that along the other two directions. CHC exhibits outstanding mechanical properties at a temperature range from 200 K to 500 K.

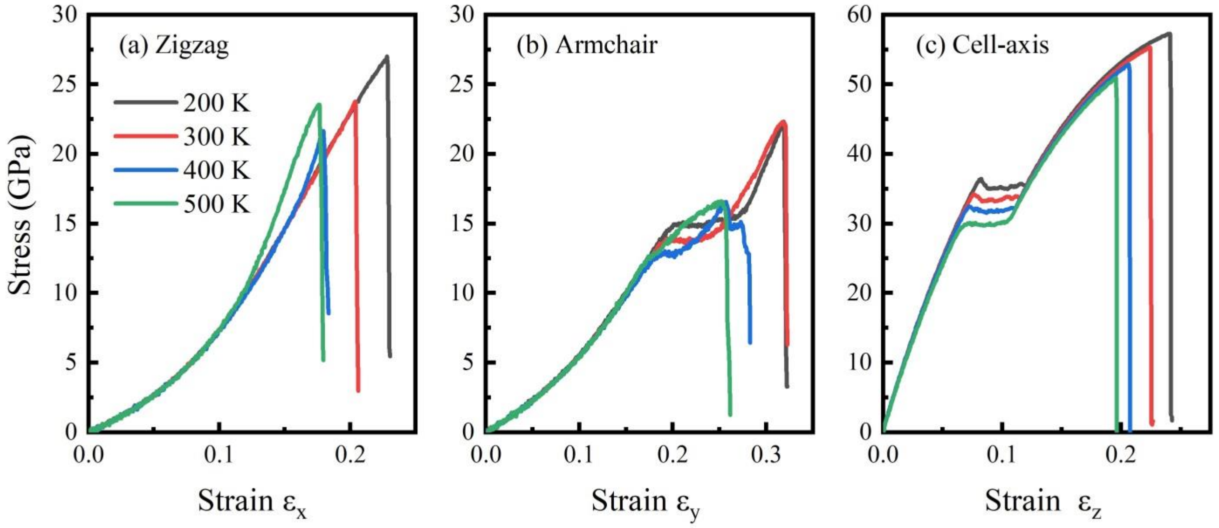

Figure S2. Engineering stress-strain curves of CHC at different temperatures when applied tensile tests along zigzag (a), armchair (b) and cell-axis (c) directions.

Supplementary Figure S3 shows the influence of temperature on the Poisson's ratio when CHC subjected to uniaxial tensile tests along cell-axis direction. In both armchair and zigzag directions, CHC exhibits near the same Poisson's ratio at different temperatures. The maximum Poisson's ratio is up to around 0.5. With increasing temperature, the maximum Poisson's ratio occurs at smaller tensile strain. And the NA transition is not observed when tensile strain $\varepsilon_z < 0.15$.

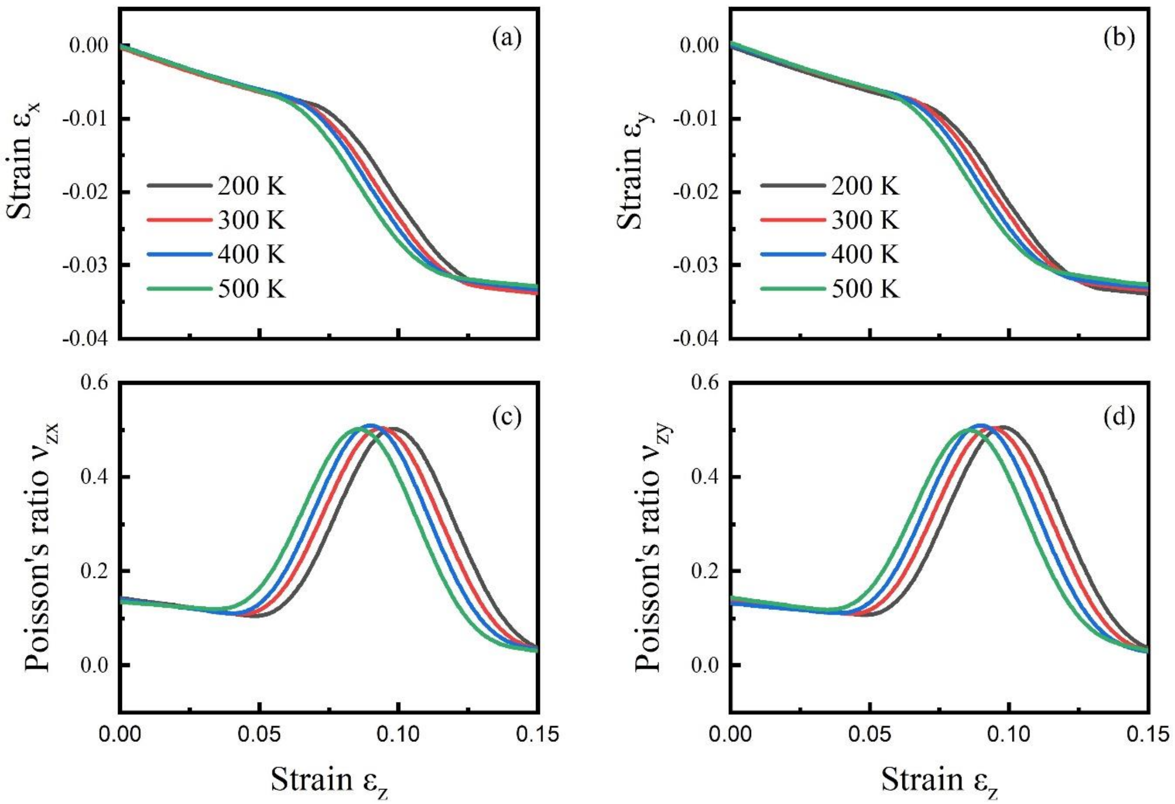

Figure S3. Temperature effect. The influence of temperature on the Poisson's ratio for carbon honeycomb subjected to uniaxial tensile tests along cell-axis (z) direction. Engineering strain ε_z as a function of ε_x (a) and ε_y (b). (c) and (d) The accompanying ν for CHC at different temperatures.

References

1. Pang, Z., et al. *Bottom-up Design of Three-Dimensional Carbon-Honeycomb with Superb Specific Strength and High Thermal Conductivity*. Nano Lett. 2017. **17**(1): p.179-85.
